# Isolation, purification and in vitro differentiation of cytotrophoblast cells from human term placenta

**DOI:** 10.1186/s12958-015-0070-8

**Published:** 2015-07-09

**Authors:** Liping Li, Danny J Schust

**Affiliations:** Department of Obstetrics, Gynecology and Women’s Health, University of Missouri School of Medicine, 500 N. Keene Street, Columbia, MO USA; Department of Obstetrics and Gynecology, Guangzhou Medical University Affiliated Guangzhou First People’s Hospital, 1 Panfu Road, Guangzhou, China

**Keywords:** Syncytiotrophoblast, Cytotrophoblast cells, Isolation, Differentiation, Fusion, Placenta

## Abstract

**Background:**

The syncytialization of cytotrophoblast cells to syncytiotrophoblast is central to human placental transport and hormone production. Many techniques for in vitro study of this process have been proposed and new investigators to the field may find the literature in the field daunting. Here, we present a straightforward and reliable method to establish this important model using modern but readily available tools and reagents.

**Methods:**

Villous cytotrophoblast cells are obtained from term placenta using mild enzymatic degradation, Percoll gradient centrifugation, negative magnetic cell sorting using an antibody against classical major histocompatibility complex molecules and in vitro culture on a matrix-coated growth surface.

**Results:**

The purity of isolated cytotrophoblast cells exceeds 98 % as assessed by cytokeratin-7 expression using flow cytometry. Contamination by mesenchymal cells, extravillous trophoblast cells, leukocytes, Hofbauer and endothelial cells is minimized (less than 2 % when analyzed for vimentin, HLA-G, CD45, CD163 and CD31 using flow cytometry). Isolated cytotrophoblast cells began to aggregate into monolayers of mononucleated cells within about 12 h of plating. By 72 h in culture, most cytotrophoblast cells have differentiated into syncytiotrophoblast as demonstrated by a loss of intercellular E-cadherin expression upon fusion into multinucleated syncytia. After 72 h in culture, nearly every cultured cell expresses syncytiotrophoblast markers, including cytokeratin-7, human chorionic gonadotropin-β (β-hCG) and the fusion-related proteins glial cell missing-1 (GCM-1) and syncytin.

**Conclusions:**

We present an efficient and reliable method for isolating of cytotrophoblast cells with high purity and complete differentiation into syncytiotrophoblast in vitro.

## Background

Placental villous syncytiotrophoblast mediates maternal-fetal transfer of nutrients critical for fetal growth and development. This highly specialized epithelium also performs a number of other functions including pregnancy hormone production and secretion [1]. The syncytiotrophoblast arises from fusion and differentiation of the underlying mitotically active cytotrophoblast cells [2]. This process is characterized by morphological and biochemical differentiation. The morphological differentiation is defined by the fusion of mononucleated cytotrophoblast cells with adjacent syncytium, while the biochemical differentiation is characterized by the production of hormones such as human chorionic gonadotropin (hCG) and human placental lactogen (hPL) [3]. The importance of syncytiotrophoblast for the progression of normal pregnancy is highlighted by the fact that its dysregulation is linked to pregnancy complications associated with maternal and/or fetal morbidity and mortality, including preeclampsia [4–6] and fetal growth restriction [7, 8].

The ability to readily and reproducibly obtain non-immortalized human cytotrophoblast cells and syncytiotrophoblast are necessary for the study of placental function and of the roles of each of these cellular components in the normal physiology and pathologies of human reproduction. Because of its syncytial structure, it is inherently difficult to directly isolate syncytiotrophoblast. Therefore, the first step in the production of cultured villous syncytiotrophoblast has been to isolate its undifferentiated in vivo precursor, villous cytotrophoblast cells.

Almost all current methods for isolation and culture of placental cytotrophoblast cells are derived from the classic work of Kilman et al, which was published in 1986 [9]. Since that time, improvements in digestion agents have allowed investigators to move away from the fairly harsh conditions of trypsin-mediated tissue digestion [10, 11], although the use of Percoll density gradients for initial isolation of appropriate cell populations post-digestion remains standard. The intervening years have also allowed the introduction of very specific tools for positive and negative antibody-mediated separation of cytotrophoblast cells from non-trophoblast-derived contaminants [12, 13]. The many permutations of these approaches that can be found in the existing literature make it difficult to choose a method for the purification of villous trophoblast cells from normal human placenta that will be reproducible, efficient and reliable.

In this study, we describe an optimized method for isolation and culture of villous trophoblast from human term placenta that meets these requirements. The methods are straightforward and reproducibly obtain cytotrophoblast cells with high purity and viability. These cells can be easily maintained under common culture conditions and will differentiate over the course of a few days into multinucleated cells that express cytokeratin-7, β-hCG and fusion proteins characteristic of syncytiotrophoblast cells. In addition to their utility in the study of placental differentiation and cytotrophoblast fusion into syncytiotrophoblast, the cultured cytotrophoblast cells and syncytiotrophoblast obtained using these methods could be used in combination with other in vitro cell models to study a variety of other aspects of placental function in vitro, including trophoblast-endometrial interactions during normal and abnormal implantation, reproductive immunology, placental endocrinology, trophoblast/endometrial growth factor and cytokine metabolism, and even basic mechanisms of cell differentiation.

## Methods

This study was approved by University of Missouri Health Sciences Institutional Review Board. The experimental procedures were performed between July 2014 and May 2015.

### Reagents

Dispase II, collagenase I, Dulbecco's phosphate-buffered saline (DPBS) without calcium and magnesium, Dulbecco’s Modified Eagle’s Medium (DMEM), penicillin and streptomycin, fetal bovine serum (FBS), bovine serum albumin (BSA), Alexa Fluor® 488 donkey anti-rabbit IgG, Alexa Fluor® 488 donkey anti-goat IgG, Alexa Fluor® 594 donkey anti-rabbit IgG and Alexa Fluor® 488 donkey anti-mouse IgG (H + L) were obtained from Life Technologies (Grand Island, NY, USA). PE-conjugated anti-HLA-ABC, anti-PE microbeads and MACS separation buffer were purchased from Miltenyi Biotec (Bergisch Gladbach, Germany). APC-conjugated anti-HLA-G, APC/Cy7-conjugated anti-CD45, PE/Cy7-conjugated anti-CD163 and matched fluorescence-labeled isotype control antibodies were obtained from BioLegend (San Diego, CA, USA). PerCP-eFluor® 710-conjugated anti-CD31 and the matched fluorescence-labeled isotype control antibody were obtained from eBioscience (San Diego, CA, USA). Alexa Fluor® 488-conjugated anti-vimentin, PE-conjugated anti-cytokeratin-7, matched fluorescence-labeled isotype control antibodies, fixation buffer, and permeabilization wash buffer were purchased from BD Biosciences (San Jose, CA, USA). Goat anti-human cytokeratin-7 and rabbit anti-human syncytin were obtained from Santa Cruz Biotechnology (Dallas, Texas, USA). Rabbit anti-human β-hCG and mouse anti-human glial cell missing-1 (GCM1) were purchased from Abcam (Cambridge, UK). Rabbit anti-human E-cadherin was purchased from Cell Signaling (Danvers, MA, USA). DNase I was obtained from Roche (Basel, Switzerland). Percoll was purchased from GE Healthcare (Pittsburgh, PA, USA). Marigel was obtained from Sigma-Aldrich (St. Louis, MO, USA). VECTASHIELD® mounting media with DAPI was purchased from Vector Laboratories (Burlingame, CA, USA).

### Isolation protocol

Human term placentas were collected from normal vaginal or cesarean deliveries performed by the faculty of the Department of Obstetrics, Gynecology and Women's Health at the Missouri Women’s and Children’s Hospital, University of Missouri School of Medicine. All samples were processed immediately following delivery. Several cubic millimeters of the basal plate surface were removed sharply. Villous tissue was gently scraped free from vessels and connective tissue using the blunt edge of a scalpel. After washing thoroughly with DPBS, the tissue was cut into small pieces. Every wet gram of minced tissue was digested three times in 5 ml of a digestion enzyme medium containing 1 mg/ml Dispase II, 0.5 mg/ml collagenase I and 0.1 mg/ml DNase I in DMEM at 37 °C for 20 min each cycle in a shaking water bath. Enzymatic degradation was stopped by adding an equal volume of complete DMEM containing 10 % FBS, 100 U/ml penicillin and 100 μg/ml streptomycin. The mix was filtered through a 100 μm strainer (Thermo Fisher Scientific, Waltham, MA, USA) and then a 40 μm strainer (Thermo Fisher Scientific) to remove contaminating tissue debris. Cell suspensions were collected and centrifuged at 350 g for 10 min at 4 °C. Cell pellets were resuspended in 5 ml of complete DMEM, layered on the top of a preformed Percoll gradient (65 %, 55 %, 50 %, 45 %, 35 %, 30 % and 25 %) and centrifuged at 730 g at 4 °C without braking for 30 min. The layer between the 45 % and 35 % Percoll aliquots containing trophoblast cells (density 1.050-1.060 g/ml) was collected, suspended in complete DMEM and centrifuged at 350 g at 4 °C for 10 min. The resulting cell pellet was resuspended in PBS, cells were counted and the suspension was re-centrifuged at 350 g at 4 °C for 10 min.

### Immunopurification of cytotrophoblast cells

Before primary culture, non-trophoblast cell contaminants were depleted using a PE-conjugated HLA-ABC antibody and anti-PE microbeads according to the manufacturer’s instructions (Miltenyi Biotec). Briefly, cell pellets were resuspended in 100 μl of MACS separation buffer per 10^7^ cells and incubated with 10 μl of PE-conjugated anti-HLA-ABC antibody for 10 min at 4 °C. After washing, the cells were resuspended in 80 μl of buffer and incubated with 20 μl of anti-PE microbeads for 15 min at 4 °C. Cell pellets were washed and resuspended in 500 μl of buffer. HLA-ABC^+^ cells were depleted using an MS column and a MiniMACS^TM^ separator (Miltenyi Biotec). The remaining trophoblast cell fraction was counted and cell viability was estimated by Trypan blue exclusion.

### Primary culture of isolated cytotrophoblast cells

Isolated cells were plated on a Matrigel-coated culture surface at a density of 4 × 10^5^ cells/cm^2^ in complete DMEM at 37 °C in 5 % CO_2_. After allowing the cells to attach for 4 h, medium and unattached cells were removed to eliminate preformed syncytial fragments that would not survive extended culture.

### Flow cytometry

Aliquots of 10^6^ cells in 50 μl DPBS were incubated with APC-conjugated anti-HLA-G (5 μl), APC/Cy7-conjugated anti-CD45 (5 μl), PE/Cy7-conjugated anti-CD163 (5 μl) or PerCP-eFluor® 710-conjugated anti-CD31 (5 μl) antibodies for 30 min at 4 °C. After washing twice with DPBS, cells were fixed in a fixation buffer. For intracellular cytokine staining, cells were fixed in a fixation buffer and incubated for 10 min at room temperature. After washing with DPBS, cells were resuspended in a permeabilization wash buffer and incubated with Alexa Fluor® 488-conjugated anti-vimentin (5 μl) and PE-conjugated anti-cytokeratin-7 (20 μl) antibodies. Isotype controls were established using matched fluorescence-labeled isotype control antibodies and equivalent immunostaining conditions. Immunostained cells were analyzed on a CyAn ADP flow cytometer (Beckman Coulter, Brea, CA, USA) using Summit 5.2 software (Beckman Coulter). The expression levels of cytokeratin-7, vimentin, CD45, CD 163, HLA-G and CD31 in purified cytotrophoblast cells were measured and graphed.

### Immunocytochemistry

Isolated primary cytotrophoblast cells were cultured for 72 h. Cells were then fixed in 4 % paraformaldehyde for 15 min at room temperature. After washing three times with DPBS, cells were permeabilized with DPBS containing 0.1 % triton for 20 min at room temperature, then incubated with DPBS containing 5 % BSA for 1 h at room temperature to block nonspecific staining. Samples were rinsed with DPBS and incubated with rabbit anti-human E-cadherin (1/100), goat anti-human cytokeratin-7 (1/100) and rabbit anti-human β-hCG (1/100), mouse anti-human GCM1 (1/100) or rabbit anti-human syncytin (1/100) in a humidified box overnight at 4 °C. Cells were then washed three times with DPBS and incubated with Alexa Fluor® 488 donkey anti-rabbit IgG (1/300), Alexa Fluor® 488 donkey anti-goat IgG (1/300) and Alexa Fluor® 594 donkey anti-rabbit IgG (1/300), Alexa Fluor® 488 donkey anti-mouse IgG (H + L) (1/300) or Alexa Fluor® 594 donkey anti-human rabbit IgG (1/300) for 1 h at room temperature. After washing three times with DPBS, the cells were mounted in VECTASHIELD® mounting media with DAPI. Samples were observed under a Leica TCP SP8 MP confocal laser-scanning microscope (Leica Microsystems, Wetzlar, Germany) using Leica LAS AF Lite software (Leica Microsystems).

### Statistical analysis

All statistical analyses were performed using SPSS 19.0 software (IBM, Armonk, NY, USA). Data were analyzed using one-way ANOVA with Bonferroni post-testing. Results were expressed as mean ± SD. A *p* value of < 0.05 was considered significant.

## Results

### Comparison of yield and viability of purified cytotrophoblast cells among diverse enzymatic digestion protocols

**Fig. 1:**
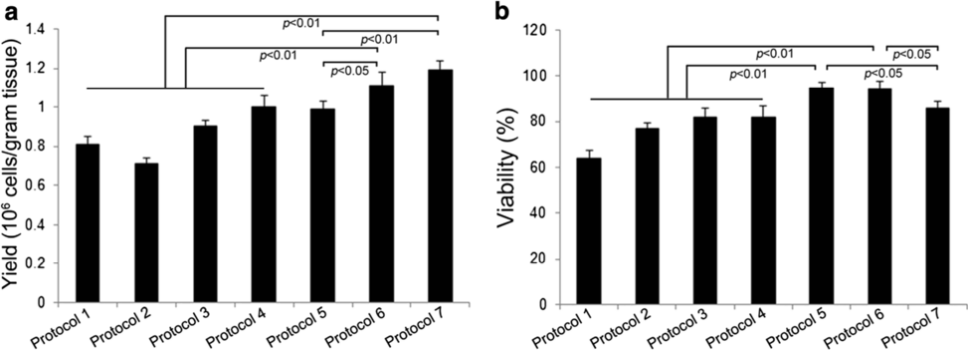
Comparison of yield and viability of purified cytotrophoblast cells among a variety of enzymatic degradation protocols. Yield (**a**) and viability (**b**) of purified cytotrophoblast cells using different enzymatic digestion protocols were assessed. Protocol 1: digestion three times in 0.25 % trypsin for 30 min each [10]; Protocol 2: digestion two times in 0.25 % trypsin for 10 min each [11]; Protocol 3: digestion in 0.125 % trypsin and 0.2 mg/ml DNase I for 45 min [14]; Protocol 4: digestion three times in 0.125 % trypsin and 0.2 mg/ml DNase I for 30 min each [15]; Protocol 5: digestion three times in 1 mg/ml Dispase II, 0.5 mg/ml collagenase I and 0.1 mg/ml DNase I for 15 min each; Protocol 6: digestion three times in 1 mg/ml Dispase II, 0.5 mg/ml collagenase I and 0.1 mg/ml DNase I for 20 min each; Protocol 7: digestion two times in 1 mg/ml Dispase II, 0.5 mg/ml collagenase I and 0.1 mg/ml DNase I for 30 min each. Data are presented as mean ± SD of six independent experiments

To determine which digestion protocol simultaneously optimized yield and viability in the isolated cytotrophoblast cells, we exposed placental tissue to trypsin alone [10, 11] or in combination with other enzymes [14, 15], as well as proteolytic enzymes other than trypsin for various incubation times. As shown in Fig. 1, three digestions for 20 min each using an enzymatic cocktail composed of dispase II, collagenase I and DNase I (Protocol 6) resulted in the best combination of yield and cell viability. Using this protocol, the average yield of purified cytotrophoblast cells was (1.11 ± 0.07) × 10^6^ cells/gram tissue and average cell viability was 94.4 % ± 3.2 % as judged by Trypan blue exclusion (*n* = 6).

### Cytotrophoblast cell purity after only Percoll isolation

**Fig. 2:**
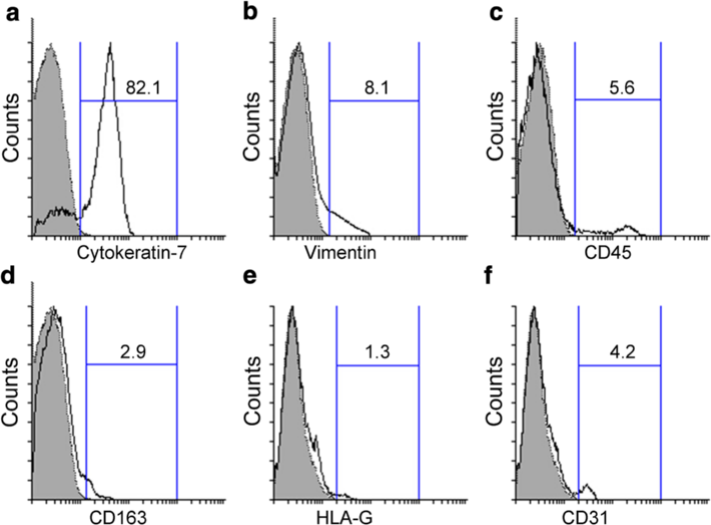
Purity of villous cytotrophoblast cells after Percoll isolation. The expression levels of cytokeratin-7 (**a**), vimentin (**b**), CD45 (**c**), CD163 (**d**), HLA-G (**e**) and CD31 (**f**) in Percoll-isolated cytotrophoblast cells were analyzed using flow cytometry. Gray shaded histogram: isotype-matched negative control. Black line: specific antibody expression. Numbers indicate the percentages of particular antibody positive cells among isolated cells (%). The depicted result is representative of four independent experiments

The purity of primary cytotrophoblast cells was analyzed based on the expression of cytokeratin-7 using flow cytometry. The percentage of cells that expressed cytokeratin-7 immediately after Percoll centrifugation was only about 80 % (Fig. 2a). Approximately 8 % of the cells expressed vimentin, a mesenchymal cell marker used to identify non-trophoblast contaminants (Fig. 2b). Contaminating leukocytes accounted for more than 5 % of the isolated cells as assessed by the expression of the pan leukocyte marker CD45 (Fig. 2c). Cells expressing CD163, a specific marker of fetal macrophages (Hofbauer cells), accounted for about 3 % of the isolated cells (Fig. 2d). Contamination by extravillous cytotrophoblast cells expressing HLA-G was slightly more than 1 % (Fig. 2e). Contaminating endothelial cells including fetal endothelial cells expressing CD31 made up more than 4 % of isolated cells (Fig. 2f).

### Purity of cytotrophoblast cells after immunopurification

**Fig. 3:**
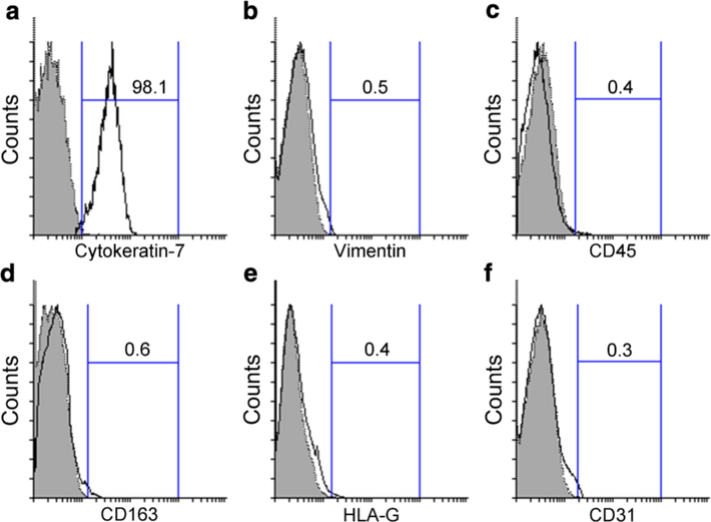
Purity of villous cytotrophoblast cells after immunopurification. The expression levels of cytokeratin-7 (**a**), vimentin (**b**), CD45 (**c**), CD163 (**d**), HLA-G (**e**) and CD31 (**f**) in immunopurified cytotrophoblast cells were analyzed using flow cytometry. Gray shaded histogram: isotype-matched negative control. Black line: specific antibody expression. Numbers indicate the percentages of particular antibody positive cells among isolated cells (%). The depicted result is representative of four independent experiments

The percentage of cytokeratin-7 positive cytotrophoblast cells exceeded 98 % after Percoll separation followed by immunopurification (Fig. 3a). Contaminating mesenchymal cells, leukocytes, Hofbauer cells, extravillous cytotrophoblast cells and endothelial cells comprised less than 2 % of these doubly purified cells (Fig. 3b-f).

### Differentiation of cytotrophoblast cells into syncytiotrophoblast in culture

**Fig. 4:**
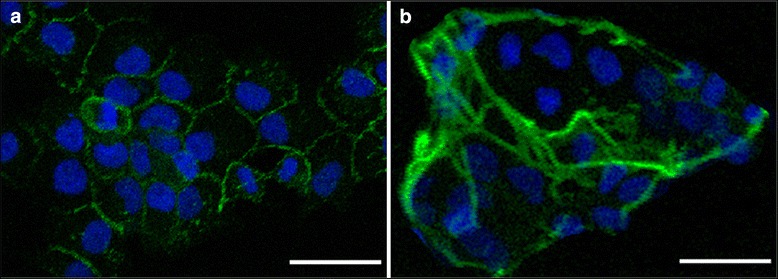
Expression of E-cadherin on isolated trophoblast cells. Primary trophoblast cells isolated from human term placentas were stained with E-cadherin (green). Nuclei were stained with DAPI (blue). Confocal microscopy, scale bar: 50 μm. **a** Mononuclear cytotrophoblast cells aggregated by 12 h in culture. **b** Most cytotrophoblast cells fused to form multinucleated syncytia by 72 h in culture

**Fig. 5:**
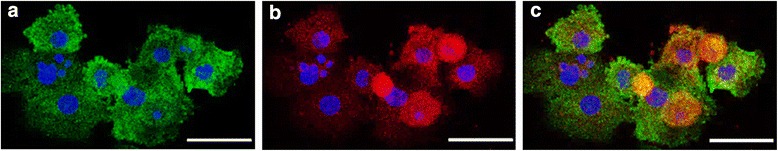
Expression of cytokeratin-7 and β-hCG in isolated trophoblast cells after 72 h in culture. **a** Primary trophoblast cells isolated from human term placentas were culture for 72 h and stained with cytokeratin-7 (green). Nuclei were stained with DAPI (blue). **b** Primary trophoblast cells isolated from human term placentas were culture for 72 h and stained with β-hCG (red). Nuclei were stained with DAPI (blue). **c** Merge of cytokeratin-7 and β-hCG immunostaining images. Confocal microscopy, scale bar: 50 μm

**Fig. 6:**
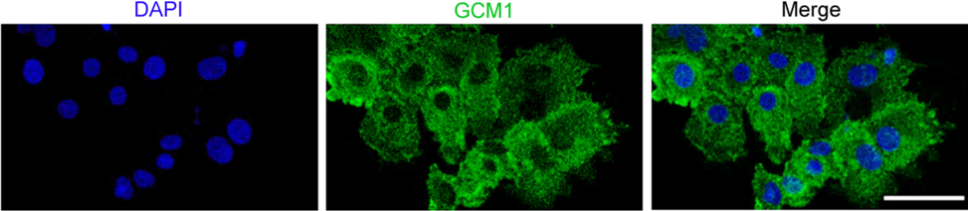
Expression of GCM1 on isolated trophoblast cells after 72 h in culture. Primary trophoblast cells isolated from human term placentas were culture for 72 h and stained with an antibody against GCM1 (green). Nuclei were stained with DAPI (blue). Confocal microscopy, scale bar: 50 μm

**Fig. 7:**
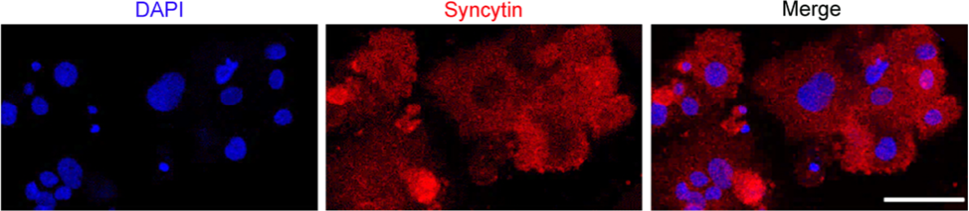
Expression of syncytin on isolated trophoblast cells after 72 h in culture. Primary trophoblast cells isolated from human term placentas were culture for 72 h and stained with an antibody against syncytin (red). Nuclei were stained with DAPI (blue). Confocal microscopy, scale bar: 50 μm

Isolated cytotrophoblast cells began to aggregate into monolayers of mononucleated cells within about 12 h of plating. Immunofluorecent staining of E-cadherin presented at the boundaries of aggregated cells (Fig. 4a). By 72 h in culture, most cytotrophoblast cells had differentiated into syncytiotroblast as demonstrated by a loss of intercellular E-cadherin expression upon fusion into multinucleated syncytia (Fig. 4b). As shown in Fig. 5, nearly every cell expressed both cytokeratin-7 and β-hCG simutaneously after 72 h of culture, visually demonstrating the presence of syncytiotrophoblast and the high purity of the isolated cells. In addition, strong staining for the fusion transcript factor GCM1 (Fig. 6) and the fusion protein syncytin (Fig. 7) could also be detected on all of the cells in culture. Together with multinuclear morphology, these expression patterns indicate the differentiation of purified cytotrophoblast cells into syncytiotrophoblast.

## Discussion

Isolation of viable mononuclear cytotrophoblast cells with high purity from placental tissues is essential to any reliable in vitro culture method that allows for subsequent differentiation into syncytiotrophoblast. Optimizing this isolation requires a delicate balance during the initial enzymatic degradation of freshly isolated tissues. In most isolation methods, trypsin is used either alone [10, 11] or in combination with other enzymes [14, 15]. However, the use of trypsin for digestion has several drawbacks. When compared to milder digestion reagents, trypsin exposure results in limited survival of isolated cells after dispersion and collection [16]. Moreover, trypsin may affect protein surface expression profiles on isolated cytotrophoblast cells and thereby complicate immunopurification and determination of cell purity. Such altered cell surface profiles may also alter subsequent functional studies of the cytotrophoblast cells and/or syncytiotrophoblast. Several proteolytic enzymes other than trypsin have been used alone or in combination to digest placental villous tissues in an effort to maximize the viability and surface protein signatures of the isolated cells, including pronase with DNase I [17], collagenase combined with DNase IV and protease XIV [18], and dispase II combined with DNase I [19].

Contamination of isolated cytotrophoblast cells by stromal or other cells is minimized when the mildest and shortest disaggregation conditions are employed. The ideal enzymatic digestion would obtain the highest possible yield and viability of the desired cells while minimizing collection of contaminating cells. In this study, we compared the effects of trypsin (alone or in combination with DNase I) and the enzymatic cocktail (dispase II, collagenase I and DNase I) under a variety of incubation times and digestion repeats in parallel experiments. We found that dispase II in conjunction with collagenase I effectively but gently released cells from placental tissue without significantly affecting cell viability. The addition of DNase I was used to reduce the viscosity of the digests and improve the efficiency of single cell separation. In addition, the use of three 20 min cycles of digestion with our enzymatic cocktail gave the highest yield without significantly compromising cell viability.

To optimize efficiency and reliability, it is important to obtain a true single-cell suspension before Percoll gradient centrifugation and subsequent magnetic cell separation. It has been previously shown that the majority of the freshly isolated cytotrophoblast cells will have a diameter of about 10 μm, although these cells may occasionally be as large as 20-30 μm in diameter [2]. In our optimized protocol, we used a 40-μm-pore strainer to eliminate tiny tissue debris and extant syncytial fragments before Percoll isolation. The density gradient is certainly useful in separating red blood cells and remaining cellular debris from the bulk of all other cells. However, use of a density gradient alone isolates a fairly crude cellular population with broad phenotypic variation and an unacceptably high level of cellular contamination by mesenchymal cells, leukocytes, Hofbauer cells and extravillous trophoblast cells (Fig. 2). Further immunopurification is essential to reliably and efficiently isolate primary cytotrophoblast cells.

To date, there appears to be no consensus regarding which specific surface marker or markers should be used to consistently isolate cytotrophoblast cells, although Pötgens et al. [20] has presented a method for isolation of villous cytotrophoblast cells based on positive immonoselection using an antibody against hepatocyte growth factor activator inhibitor 1 (HAI-1) and magnetic separation. However, positive selection has a potential disadvantage in that cytotrophoblast cells may become activated during the isolation process, perhaps complicating subsequent functional studies. Others have used immunomagnetic microspheres coated with antibodies against specific antigens on the cell surface to deplete cell harvest of non-trophoblastic cells before primary culture. Douglas and King [13] were the first to report the use of such negative immunopurification, employing magnetic microspheres coated with antibodies against HLA class I (anti-HLA-ABC) and HLA class II. Others have depleted CD45^+^ cells [21], HLA-ABC^+^ cells and CD45^+^ cells [22], CD9^+^ cells and CD45^+^ cells [23], CD45^+^ cells and HLA class II^+^ cells [12], CD3^+^ cells, CD49^+^ cells, CD31^+^ cells, CD14^+^ cells and CD45RA^+^ cells [24]. The reaction patterns of marker antibodies for non-trophoblastic cells are listed in Table 1.

**Table 1:** Reaction pattern of marker antibodies for non-trophoblastic cells

Antigen	CT	ST	EVT	Leukocytes	Mesenchymal cells			References
					Fibroblasts, myofibroblasts, smooth muscle cells	Endothelial cells	Hofbauer cells	
*CD9*	-	-	+	+	+	+	-	[28, 29]
*CD45*	-	-	-	+	-	-	+	[30]
*FSA*	-	-	-	-	+	+	-	[31]
*HLA-I*	-	-	+	+	+	+	+	[25]
*HLA-G*	-	-	+	-	-	-	-	[32]
*HLA-II*	-	-	-	+	-	-	+	[12]
*Vimentin*	-	-	-	+	+	+	+	[31]
*CD163*	-	-	-	-	-	-	+	[23]

Abbreviations: FSA, fibroblast specific antigen; CK7, cytokerain-7; CT, cytotrophoblast cells; ST, syncytiotrophoblast; EVT, extravillous cytotrophoblast cells

HLA-ABC antigens are expressed by all human nucleated cells except villous cytotrophoblast cells and derivative syncytiotrophoblast. As shown in Table 1, using beads-coated anti-HLA-ABC antibody is sufficient to effectively deplete non-trophoblastic cells during cytotrophoblast cell isolation. Since each step of immunodepletion inevitably decreases the overall yield of non-selected cells as well as those reacting to the antibody bound to the microbead we chose to include a single immunopurification step in our final protocol.

Syncytial fragments do not attach to most growth surfaces and these can be removed by careful washing after 4 h of culture [25]. The aggregation of villous cytotrophoblast cells, a necessary step before the cells fuse to form syncytia, requires movement across the culture surface to make contact with each other. A protein extracellular matrix culture surface helps to allow cellular movement [3]. In this study, we used Matrigel to improve cytotrophoblast cell aggregation and syncytialization.

The purity of isolated trophoblast cells and the nature of contaminating cells should be assessed carefully when working with these primary in vitro culture models. If the intention of the planned investigation is the study of trophoblast cell differentiation, cytotrophoblast cell isolation must aim for a highly purified yield. Assessment of purity using flow cytometry is both objective and quantitative. Here we evaluated the expression of the intracellular marker cytokeratin-7 to check the purity of isolated trophoblast cells since this trophoblast-specific marker has been shown to be an accurate and reliable marker for the identification of human villous trophoblast cells obtained from both early and term placenta [24]. In our study, more than 98 % of purified cells expressed cytokeratin-7. High purity was confirmed by immunostaining with cytokeratin-7. Conversely, we assessed the presence of contaminating cells using flow cytometry and antibodies against the mesenchymal marker, vimentin (expressed in fibroblasts, myofibroblasts, smooth muscle cells and endothelial cells), the extravillous cytotrophoblast cell marker, HLA-G, the pan-leukocyte marker, CD45, the Hofbauer cell marker, CD163 and the endothelial cell marker, CD31. The total percentage of these non-villous cytotrophoblast cell contaminants in our preparations was less than 2 %.

Immunostaining with an anti-E-cadherin antibody delineates surface membranes and allows distinction between fused, multinucleated syncytintrophoblast and mononucleated cytotrophoblast cells. Cytotrophoblast cells isolated from human term placenta were mononuclear. Although these cells aggregated in culture within 12 h after plating, they maintained distinct intercellular borders. Most of these cells fused into multinucleated cells after 72 h in culture as assessed by morphology. To further assess the in vitro differentiation of isolated cytotrophoblast cells into syncytiotrophoblast, we used immunocytochemistry to demonstrate the simultaneous expression of cytokeratin-7 and β-hCG and confocal microscopy. Although the presence of hormone-secreting extravillous trophoblast cells that do not form syncytia has been shown [26], we demonstrated using flow cytometry that the presence of contaminating HLA-G positive extravillous trophoblast cells in our highly purified villous cytotrophoblast cell populations was very limited. Nearly all of the purified villous cytotrophoblast cells differentiated into syncytiotrophoblast over 72 h in culture when analyzed for the expression of cytokeratin-7 and β-hCG.

Trophoblast fusion in the placenta is essential to the overall health of mother and child in humans. This process may extend well into the third trimester of pregnancy, but is initiated at the very earliest stages of human gestation. Abnormalities in this process during implantation and the first several weeks of pregnancy cannot be studied in vivo for ethical and logistical reasons, but it may be these abnormalities that act as underpinnings of several diseases of abnormal placentation, including preeclampsia, fetal growth restriction and early pregnancy loss of euploid embryos. Several regulatory proteins have been shown to be involved in trophoblast fusion. For example, syncytin is encoded by a human endogenous retrovirus (HERV)-W element and plays a central role in trophoblast fusion [15]. GCM1 is a placenta-specific transcription factor that promotes syncytin mRNA expression [27]. Almost all of the trophoblast cells in culture expressed GCM1 and syncytin, further confirming differentiation of isolated villous cytotrophoblast cells into syncytiotrophoblast.

## Conclusions

We present here an optimized method for the isolation of villous cytotrophoblast cells from chorionic villi obtained from human term placenta. The isolation yields cells of high purity and viability that readily and spontaneously differentiate into syncytiotrophoblast in culture.

## Abbreviations

BSA: Bovine serum albumin
DMEM: Dulbecco’s modified eagle’s medium
DPBS: Dulbecco's phosphate-buffered saline
FBS: Fetal bovine serum
GCM1: Glial cell missing-1
HAI-1: Hepatocyte growth factor activator inhibitor 1
hCG: Human chorionic gonadotropin
HERV: Human endogenous retrovirus
hPL: Human placental lactogen

## References

[1] Díaz P, Wood AM, Sibley CP, Greenwood SL (2014). Intermediate conductance Ca^2+^-activated K^+^ channels modulate human placental trophoblast syncytialization. PLoS One..

[2] Kliman HJ, Segel L (2003). The placenta may predict the baby. J Theor Biol..

[3] Bloxam DL, Bax BE, Bax CM (1997). Culture of syncytiotrophoblast for the study of human placental transfer. Part II: Production, culture and use of syncytiotrophoblast. Placenta.

[4] Zhuang XW, Li J, Brost BC, Xia XY, Chen HB, Wang CX (2014). Decreased expression and altered methylation of syncytin-1 gene in human placentas associated with preeclampsia. Curr Pharm Des..

[5] Anton L, Brown AG, Bartolomei MS, Elovitz MA (2014). Differential methylation of genes associated with cell adhesion in preeclamptic placentas. PLoS One..

[6] Ruebner M, Strissel PL, Langbein M, Fahlbusch F, Wachter DL, Faschingbauer F (2010). Impaired cell fusion and differentiation in placentae from patients with intrauterine growth restriction correlate with reduced levels of HERV envelope genes. J Mol Med (Berl).

[7] Weiss G, Goldsmith LT, Taylor RN, Bellet D, Taylor HS (2009). Inflammation in reproductive disorders. Reprod Sci..

[8] Hiden U, Ghaffari-Tabrizi N, Gauster M, Tam-Amersdorfer C, Cetin I, Dieber-Rotheneder M (2013). Membrane-type matrix metalloproteinase 1 regulates trophoblast functions and is reduced in fetal growth restriction. Am J Pathol..

[9] Kliman HJ, Nestler JE, Sermasi E, Sanger JM, Strauss JF (1986). Purification, characterization, and in vitro differentiation of cytotrophoblasts from human term placentae. Endocrinology..

[10] Williams SF, Fik E, Zamudio S, Illsley NP (2012). Global protein synthesis in human trophoblast is resistant to inhibition by hypoxia. Placenta..

[11] Díaz-Luján C, Triquell MF, Schijman A, Paglini P, Fretes RE (2012). Differential susceptibility of isolated human trophoblasts to infection by Trypanosoma cruzi. Placenta..

[12] Kaplan A, Chung K, Kocak H, Bertolotto C, Uh A, Hobel CJ (2008). Group B streptococcus induces trophoblast death. Microb Pathog..

[13] Douglas GC, King BF (1989). Isolation of pure villous cytotrophoblast from term human placenta using immunomagnetic microspheres. J Immunol Methods..

[14] Linton EA, Rodriguez-Linares B, Rashid-Doubell F, Ferguson DJ, Redman CW (2003). Caveolae and caveolin-1 in human term villous trophoblast. Placenta..

[15] Sun LZ, Yang NN, De W, Xiao YS (2007). Proteomic analysis of proteins differentially expressed in preeclamptic trophoblasts. Gynecol Obstet Invest..

[16] Stenqvist AC, Chen T, Hedlund M, Dimova T, Nagaeva O, Kjellberg L (2008). An efficient optimized method for isolation of villous trophoblast cells from human early pregnancy placenta suitable for functional and molecular studies. Am J Reprod Immunol..

[17] Khan S, Katabuchi H, Araki M, Nishimura R, Okamura H (2000). Human villous macrophage-conditioned media enhance human trophoblast growth and differentiation in vitro. Biol Reprod..

[18] Sotnikova N, Voronin D, Antsiferova Y, Bukina E (2014). Interaction of decidual CD56^+^ NK with trophoblast cells during normal pregnancy and recurrent spontaneous abortion at early term of gestation. Scand J Immunol..

[19] Singh AT, Dharmarajan A, Aye IL, Keelan JA (2012). Sphingosine-sphingosine-1-phosphate pathway regulates trophoblast differentiation and syncytialization. Reprod Biomed Online..

[20] Pötgens AJ, Kataoka H, Ferstl S, Frank HG, Kaufmann P (2003). A positive immunoselection method to isolate villous cytotrophoblast cells from first trimester and term placenta to high purity. Placenta..

[21] Sengupta J, Khan MA, Huppertz B, Ghosh D (2011). In-vitro effects of the antimicrobial peptide Ala8,13,18-magainin II amide on isolated human first trimester villous trophoblast cells. Reprod Biol Endocrinol..

[22] Salomon C, Kobayashi M, Ashman K, Sobrevia L, Mitchell MD, Rice GE (2013). Hypoxia-induced changes in the bioactivity of cytotrophoblast-derived exosomes. PLoS One..

[23] Tang Z, Tadesse S, Norwitz E, Mor G, Abrahams VM, Guller S (2011). Isolation of hofbauer cells from human term placentas with high yield and purity. Am J Reprod Immunol..

[24] Maldonado-Estrada J, Menu E, Roques P, Barré-Sinoussi F, Chaouat G (2004). Evaluation of Cytokeratin 7 as an accurate intracellular marker with which to assess the purity of human placental villous trophoblast cells by flow cytometry. J Immunol Methods..

[25] Cervar-Zivkovic M, Stern C, Kay HH, Nelson DM, Wang Y (2011). Trophoblast isolation and culture. The placenta: from development to disease.

[26] Handschuh K, Guibourdenche J, Cocquebert M, Tsatsaris V, Vidaud M, Evain-Brion D (2009). Expression and regulation by PPARγ of hCG α- and β-subunits: comparison between villous and invasive extravillous trophoblastic cells. Placenta..

[27] Huppertz B, Bartz C, Kokozidou M (2006). Trophoblast fusion: fusogenic proteins, syncytins and ADAMs, and other prerequisites for syncytial fusion. Micron..

[28] Schmon B, Hartmann M, Jones CJ, Desoye G (1991). Insulin and glucose do not affect the glycogen content in isolated and cultured trophoblast cells of human term placenta. J Clin Endocrinol Metab..

[29] Hirano T, Higuchi T, Ueda M, Inoue T, Kataoka N, Maeda M (1999). CD9 is expressed in extravillous trophoblasts in association with integrin α_3_ and integrin α_5_. Mol Hum Reprod..

[30] Boyd AW (1987). Human leukocyte antigens: an update on structure, function and nomenclature. Pathology..

[31] Blaschitz A, Weiss U, Dohr G, Desoye G (2000). Antibody reaction patterns in first trimester placenta: implications for trophoblast isolation and purity screening. Placenta..

[32] Gonen-Gross T, Goldman-Wohl D, Huppertz B, Lankry D, Greenfield C, Natanson-Yaron S (2010). Inhibitory NK receptor recognition of HLA-G: regulation by contact residues and by cell specific expression at the fetal-maternal interface. PLoS One..

